# Supplementary figures and images for: A second-generation molecular clamp stabilised bivalent candidate vaccine for protection against diseases caused by respiratory syncytial virus and human metapneumovirus
Source: PLoS Pathog. 2025 Jul 17;21(7):e1013312. doi: 10.1371/journal.ppat.1013312 (PMC12270161; doi:10.1371/journal.ppat.1013312)

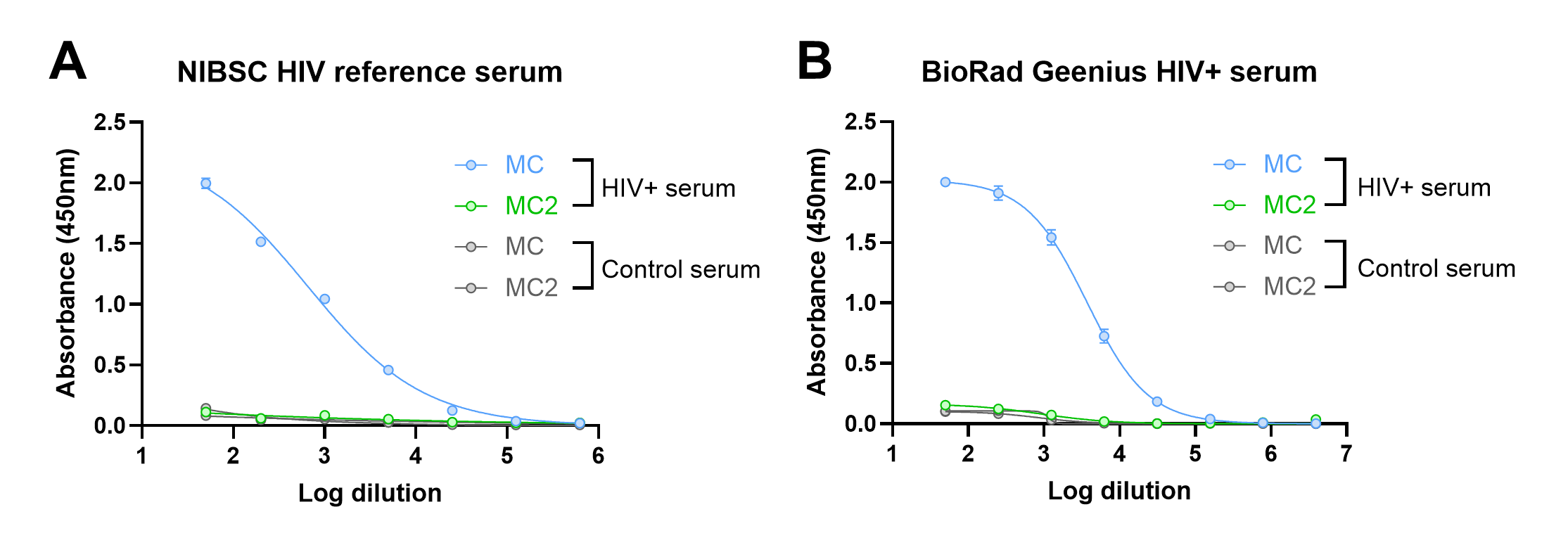

Supplement: S1 Fig — (A) NIBSC Reference serum (NIBSC code: 02/210). (B) BioRad Geenius HIV+ Serum (catalogue number #72460). (TIF) [file ppat.1013312.s001.tif]

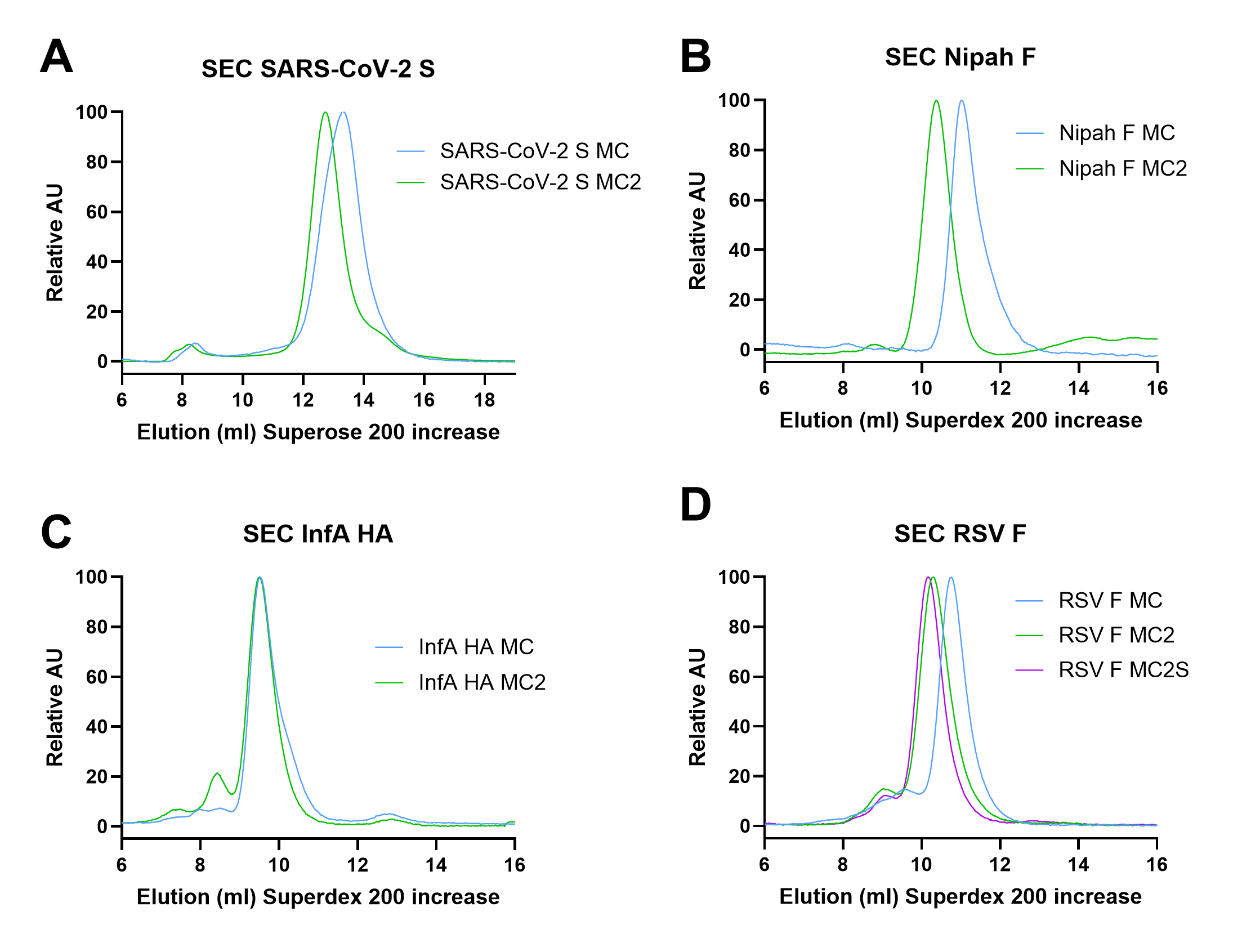

Supplement: S2 Fig — (A) SARS-CoV-2 S MC and SARS-CoV-2 S MC2 assessed with a Superose 6 Increase 10/300GL column. (B) Nipah F MC and Nipah F MC2 assessed with a Superdex 200 Increase 10/300GL column. (C) Influenza A HA MC and Influenza A HA MC2 assessed with a Superdex 200 Increase 10/300GL column. (D) RSV F MC, RSV F MC2 and RSV F MC2S assessed with a Superdex 200 Increase 10/300GL column. Note: slight differences in elution volumes are likely due to inter-run variation and column compaction. (TIF) [file ppat.1013312.s002.tif]

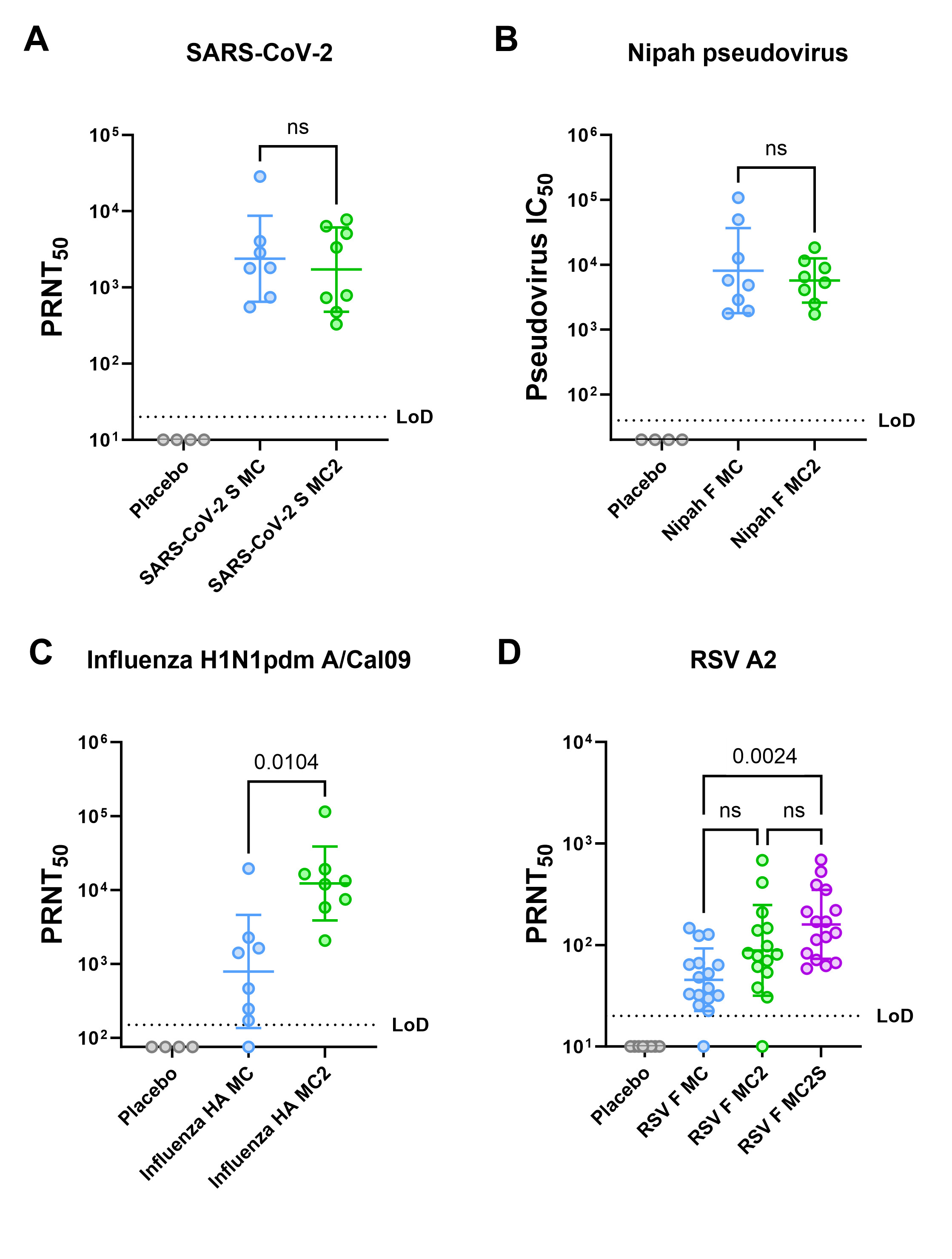

Supplement: S3 Fig — Two intramuscular doses were administered to BALB/c mice three weeks apart with each 50µl dose containing either 5µg (SARS-CoV-2 S, Nipah F, Influenza HA), or 1µg (RSV F) of purified protein in PBS and 25µl of AddaVax (InvivoGen). Three weeks following the second dose, blood serum was collected for analysis of virus neutralisation. (A) SARS-CoV-2 prototypic Wuhan strain with D614G mutation assessed by PRNT50 assay. (B) Nipah virus neutralizing titre assessed by lentivirus-based pseudoparticle assay. (C) Influenza H1N1pdm A/Cal09 neutralizing titre assessed by PRNT50 assay. (D) RSV A2 neutralizing titre assessed by PRNT50 assay Statistical analysis comparing neutralization between MC and MC2 by Mann-Whitney test (A-C), and statistical analysis comparing neutralization between MC, MC2 and MC2S, by Dunn’s multiple comparisons test (D). Bars represent geometric means + /- Standard deviation. (TIF) [file ppat.1013312.s003.tif]

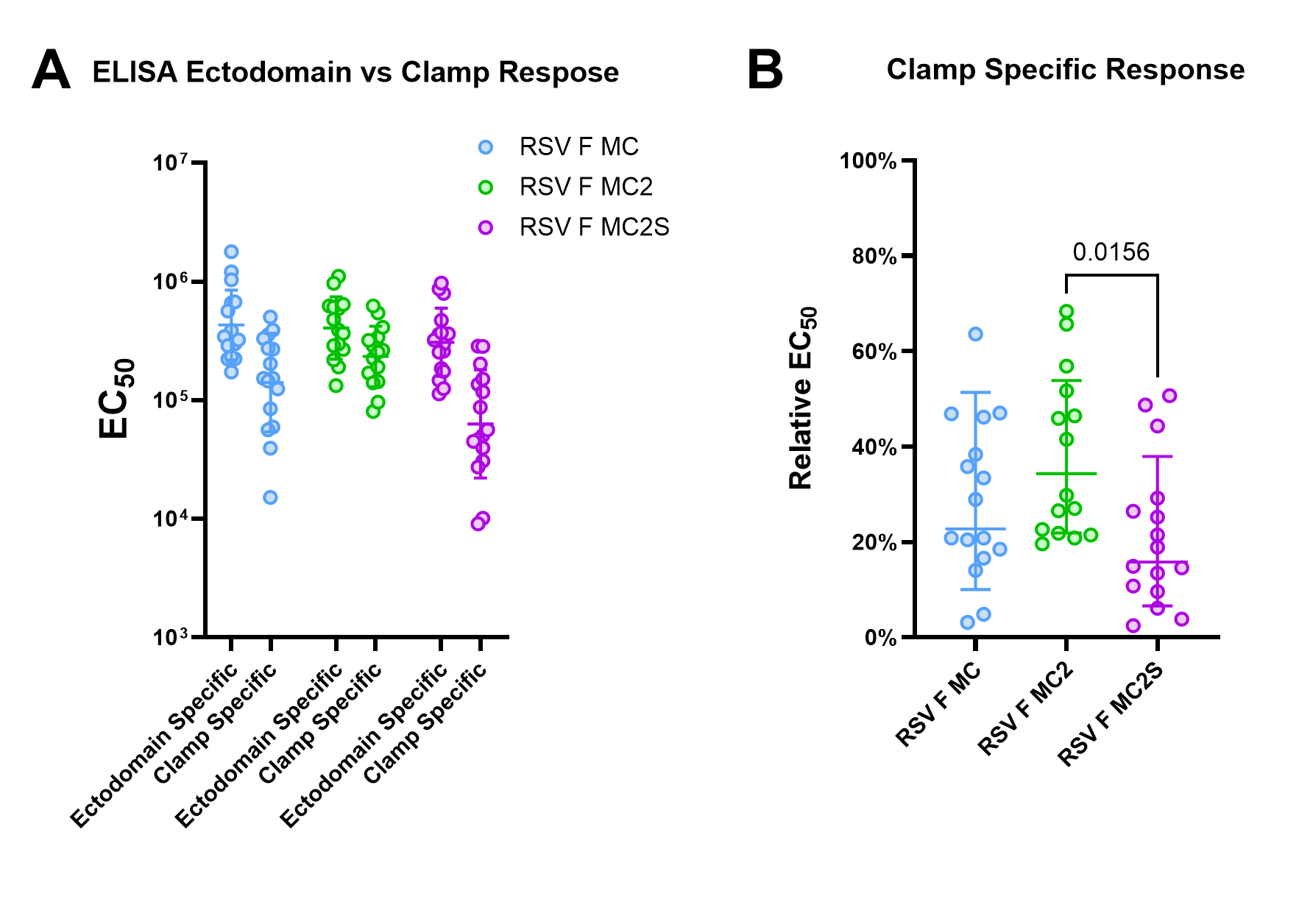

Supplement: S4 Fig — (A) ELISA EC50 values. (B) Relative percentage of IgG specific to clamp subdomain calculated via the formular EC50 clamp subdomain (MC, MC2 or MC2S) divided by total elicited IgG response (EC50 clamp subdomain plus EC50 RSV F ectodomain). Bars represent geometric means + /- Standard deviation. ANOVA Dunn’s multiple comparisons test with only significant values >0.05 shown. (TIF) [file ppat.1013312.s004.tif]

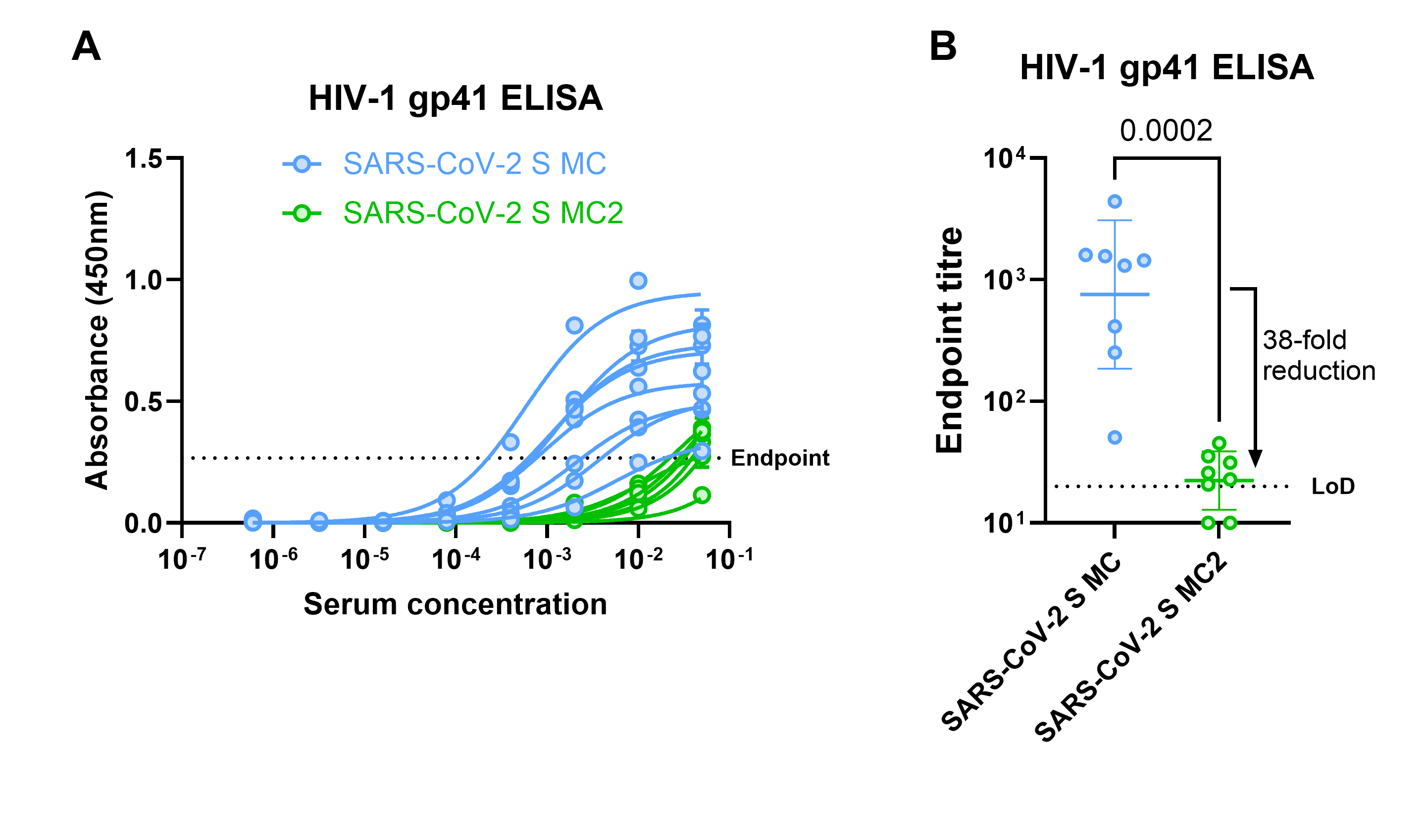

Supplement: S5 Fig — (A) ELISA readings. (B) Relative endpoints. Bars represent geometric means + /- Standard deviation. A Mann Witney test used for statistical significance. (TIF) [file ppat.1013312.s005.tif]

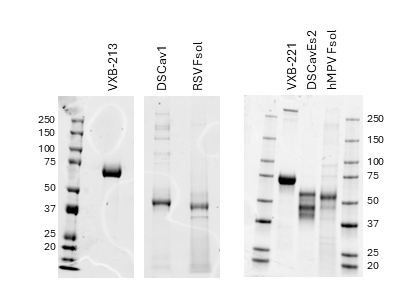

Supplement: S6 Fig — The antigens (5µg) were analysed by SDS-PAGE. All were run under reducing conditions, mixed with 100mM of dithiothreitol and 4X Laemmli sample buffer (BioRad) and boiled for 5 minutes. Gels were stained with Coomassie Brilliant Blue R-250 (BioRad) and destained using 50% RO water with 40% methanol and 10% acetic acid. To estimate the size of the denatured proteins, a Kaleidescope molecular weight ladder (BioRad) was included. (TIF) [file ppat.1013312.s006.tif]

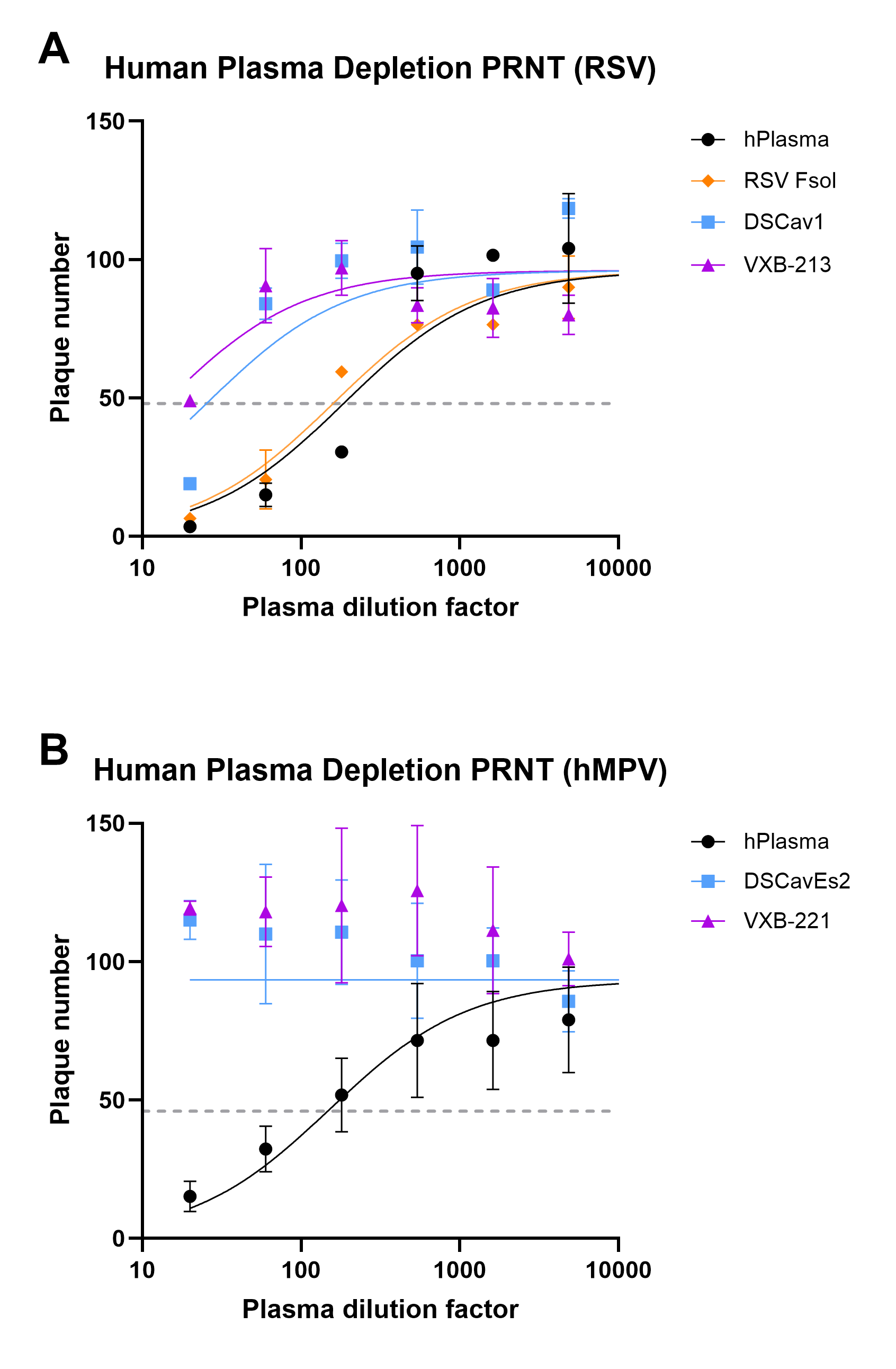

Supplement: S7 Fig — (A) RSV PRNT50 with titration of pooled human plasma (black) and human plasma pre-incubated with 0.5 µg/mL of RSV Fsol (orange), DSCav1 (blue), or VXB-213 (purple). (B) hMPV PRNT50 with titration of pooled human plasma (black) or human plasma depleted of antigen-reactive antibodies via incubation with Sepharose immobilised antigens DsCavEs2 (blue) and VXB-221 (purple). Analysis was performed using GraphPad Prism version 10.0.2 for Windows, GraphPad Software, San Diego, California USA, www.graphpad.com. (TIF) [file ppat.1013312.s007.tif]

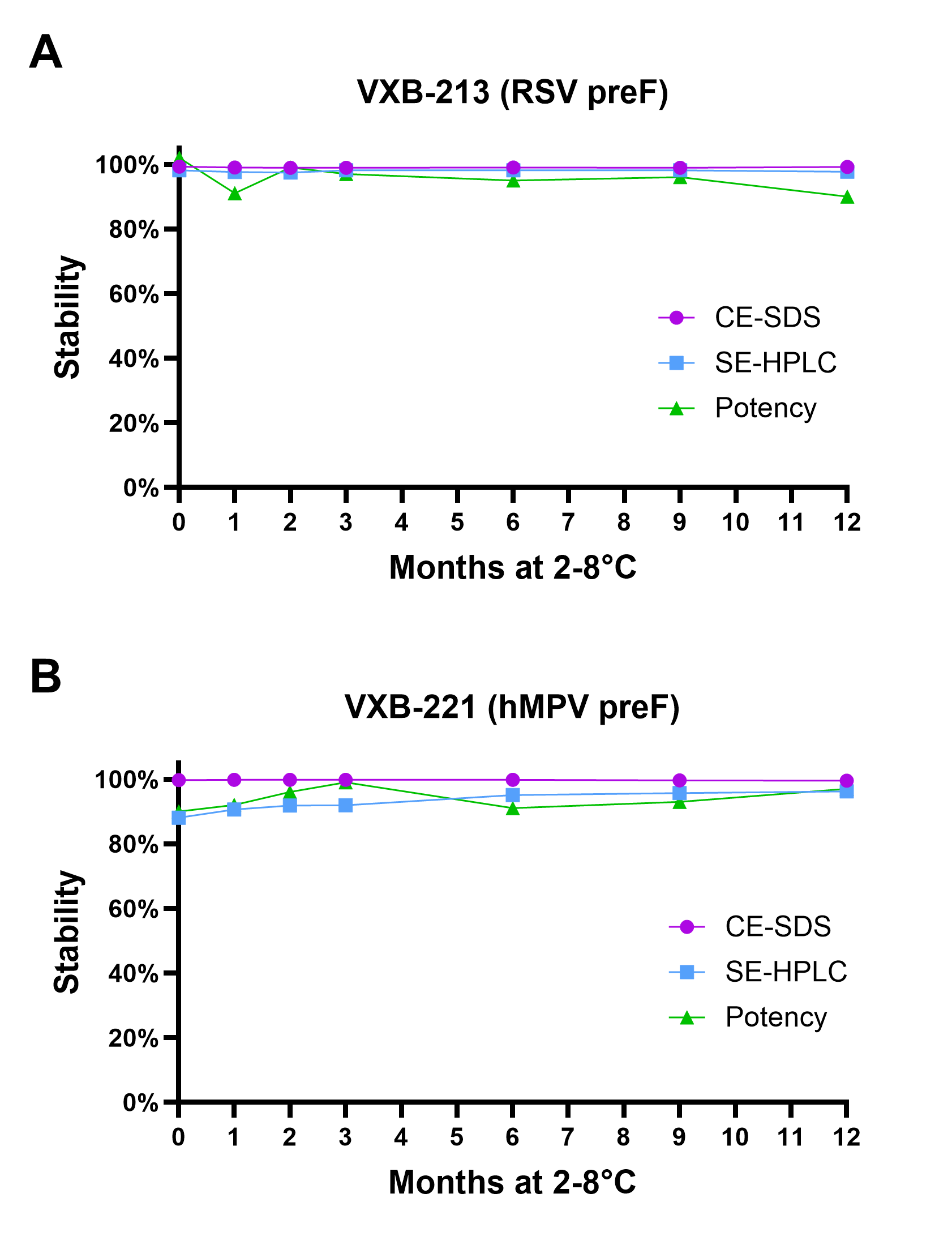

Supplement: S8 Fig — (A/B) VXB-213 and VXB-221 Stability analysis out to 12 months at 2–8°C as determined by percentage of product as a single MW by reduced CE-SDS, SE-HPLC, or by potency testing capture ELISA with paired neutralising antibodies. (TIF) [file ppat.1013312.s008.tif]

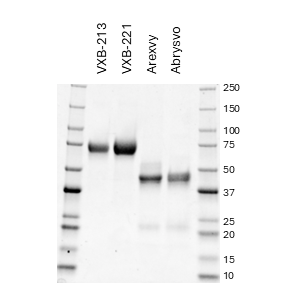

Supplement: S9 Fig — The antigens (4 µg) were analysed by SDS-PAGE. All were run under reducing conditions, mixed with 100 mM of dithiothreitol and 4X Laemmli sample buffer (BioRad) and boiled for 5 minutes. Gels were stained with Coomassie Brilliant Blue R-250 (BioRad) and destained using 50% RO water with 40% methanol and 10% acetic acid. To estimate the size of the denatured proteins, a Kaleidescope molecular weight ladder (BioRad) was included. (TIF) [file ppat.1013312.s009.tif]
