# Supplementary material for: A second-generation molecular clamp stabilised bivalent candidate vaccine for protection against diseases caused by respiratory syncytial virus and human metapneumovirus
Source: PLoS Pathog. 2025 Jul 17;21(7):e1013312. doi: 10.1371/journal.ppat.1013312 (PMC12270161; doi:10.1371/journal.ppat.1013312)
Supplement: Attachment — Submitted filename: response to reviewers.docx [file ppat.1013312.s011.docx]

Dear Editor,

We would like to thank the reviewers for their comprehensive feedback of the article. We have now incorporated the suggested changes into the manuscript and have included our response to each point raised below. We believe that these changes have improved the quality of the manuscript.

Please let us know if anything remains unclear or if further information is needed.

Many thanks,

Keith

**Major Issues: Key Experiments Required for Acceptance**
Please use this section to detail the key new experiments or modifications of existing experiments that should be absolutely required to validate study conclusions.

Generally, there should be no more than 3 such required experiments or major modifications for a "Major Revision" recommendation. If more than 3 experiments are necessary to validate the study conclusions, then you are encouraged to recommend "Reject".

Reviewer #1: 1. The authors state that their antigens are stabilized in the prefusion conformation due to the addition of MC2S (abstract: “Here we describe the design and characterisation as well as pre-clinical development of a bivalent vaccine, VXB-241, comprised of the recombinantly expressed viral fusion proteins from both RSV and hMPV, stabilised in their pre-fusion conformation by a second-generation molecular clamp (MC2S)”). However, the lead antigens all contain stabilizing amino acid substitutions described previously in the literature, including disulfide bonds. This is later clarified in the introduction, lines 146-150. Statements indicating that the clamp stabilizes the antigens in the prefusion conformation should be removed unless supporting data are provided.

We accept the point that stabilization in the prefusion conformation is substantively contributed by the point mutations and that the additional contribution of MC2S to such stabilisation has not been separately demonstrated. We have modified the text to remove the use of the phrase ‘MC2S stabilised…’ in the following places; abstract line 49, results line 187, 224, discussion line 465, 547.

2. Parts of the discussion read like an advertisement, such as the sentence “Here, we have demonstrated that the Molecular Clamp (MC) platform has significant potential for the development of a novel type of subunit vaccines offering several key advantages such as enhanced stability, improved immune responses, and the versatility to target diverse viral pathogens.” However, the authors have not directly demonstrated any advantage of the MC platform relative to other trimerization motifs. The lead antigens all contain stabilizing substitutions, which are required to stabilize the prefusion conformation. And at no point do the authors test a construct with identical stabilizing substitutions and different trimerizations motifs (MC2S vs Foldon, for instance). Therefore, statements regarding the advantages of the clamp should be considerably toned down, including “A transformative feature of MC2S…”.

We accept the point the reviewer is making hereand have modified the claims within the discussion as follows:

Line 455-45833 ‘Here, we have demonstrated that the Molecular Clamp (MC) platform has significant potential for the development of a novel type of subunit vaccines offering several key advantages such as enhanced stability, improved immune responses, and the versatility to target diverse viral pathogens.’

Changed to: ‘Here, we have demonstrated that vaccine antigens produced with the Molecular Clamp (MC) platform offer key advantages such as liquid stability and improved immune responses. The MC platform provides the versatility to target diverse viral pathogens.’

These claims are all supported by data. Liquid stability for 12 months is demonstrated (supplementary figure 8). Superior immunogenicity in terms of neutralising immune response of VXB-213 relative to Arexvy is demonstrated in figure 3 and for VXB-221 relative to DSCavEs2 is demonstrated in figure 2 (although we accept that we cannot exclue the possibility that the DSCavEs2 produced in house is different to that produced by others). Applicability to 5 viruses is shown including RSV, hMPV, SARS-CoV2, Nipah and Influenza A.

Discussion line 470-471 changed from, ‘A transformative feature of MC2S is the high production yield with a universal purification system (AVI-8740 affinity resin), which facilitates rapid advancement through pre-clinical development.’

‘The MC2S platform provides high production yield and purity with a universal purification system (AVI-8740 affinity resin), which facilitates rapid advancement through pre-clinical development.’

3. The authors claim that they use DS-CavEs2 as a comparator for hMPV F, but they do not. DS-CavEs2 contains four disulfide bonds (L110C-N322C, T365C-V463C, T127C-N153C, A140C-A147C) along with substitutions A185P, V231I, L219K, and E453Q. However, the “DS-CavEs2” construct used by the authors (as defined in Table 1) contains only two disulfide bonds (365-463 and 127-153) along with A185P, L219K, and V231I. This construct is referred to as DS-CavEs in Hsieh et al 2022: “Furthermore, the variant containing all beneficial substitutions (DSx2/L219K/V231I), exhibited an additional 1.8-fold increase compared to DSx2 and had a Tm of 60.7 °C (Fig. 3d). We renamed this variant DS-CavEs…”. The authors should either test DS-CavEs2 or modify the text accordingly.

We have indeed tested DS-CavEs2 as a comparator. The reviewer’s comment prompted us to go have gone back to our primary sequence data and confirmed that the correct sequence for DSCavEs2 was used. The mistake the reviewer identified was accidentally introduced during drafting of table 1 for the document. We apologize for this error in drafting the manuscript. Thank you for bringing this to our attention.

4. Figure 1E and Supplementary Figure 6: an explanation is needed for why DsCavEs2’s SEC trace and SDS-PAGE gel image are substantially different from the Hsieh et al, 2022 publication. Additionally, antigen QC data—such as thermostability and electron microscopy data—should be provided and compared with previous literature to confirm proper expression and folding of the antigens. A detailed protein purification method description, including affinity column types, brand names, catalog numbers, and buffer components, is also needed in the Method section.

There are 3 main method differences between our work and that of Hseih et al in the production of DSCavEs2; Hseih et al., used FreeStyle 293 cells and also cotransfected Furin during expression to facilitate cleavage of F0 in to F1 and F2. Hseih et al., also used strep tag purification followed by size exclusion chromatography (SEC) to obtain highly trimeric material. We have used CHO cells, without transfection of additional Furin and did not use size exclusion chromotography. These differences, particularly the absence of Furin overexpression likely results in the incomplete cleavage of DSCavEs2 as observed in supplementary figure 6, and the absence of SEC step to specifically isolate the trimer, likely results in the percentage of monomeric protein observed in figure 1.

To address this issue we have added these details to the results and discussion to make clear that these differences may have impacted the neutralising immune response in mice.

Results line 242-244, *‘This observation differs from the results reported by Hsieh et al., ^23^, where hMPV DSCavEs2 was expressed in Freestyle 293-F cells, in the presence of co-transfected Furin and an additional SEC purification step.’*

Discussion line 527-529, *‘… however it should be noted that differences in purification methodologies used here and those used by Hseih et al., ^23^ may have contributed to this difference. ‘*

Additional details have been added to the methods section line 616-622, *‘Immunoaffinity columns were prepared by chemical coupling of mAbs to NHS-Activated Sepharose Resing (Cytiva) using manufacturer’s instructions. Antigens were purified using high pH elution from immunoaffinity resin as previously described ^31,32^. Affinity purification of MC2S containing antigens was conducted by pre-equilibrating resin with 50mM Tris, 150mM NaCl, pH 7.4. The column was then washed with 50mM Tris, 600mM NaCl, pH 7.4 and eluted in 50mM Tris, 1M MgCl_2_, pH7.4, before then being buffer exchanged into PBS pH 7.4.’*

5. Figure 3: Statistical analyses need to be added to the plots. Furthermore, for hMPV large-scale expression, the authors should incorporate previously established controls, such as DsCavEs2, as a reference.

Additional statistical analyses have been added to figure 3

Given the cost associated with cell line development and large-scale manufacture, it is not feasible to express comparator antigens at this scale.

6. Supplementary Figures 4 and 5 require statistical analyses to support the claim that MC2S induces immune silencing compared to MC2, with p-values and test details clearly presented. Additionally, the Methods section must describe the protein expression and purification procedures for the molecular clamps used in Fig S4 and HIV-1 gp41 used in Fig S5.

Statistical analysis added as well as additional methods.

Reviewer #2: The major issue with this paper is the “best-in-class comparator antigens”. For RSV, the DSCav1 was developed more than a decade ago and is known to have less than optimal immunogenicity and stability (with significantly lower immunogenicity and stability than the 2nd-generation immunogens described by Joyce et al. 2016 NSMB); furthermore, DSCav1 fails when delivered by mRNA (Aliprantis et al. 2021 Hum Vaccin Immunother.) but succeeds when the additional stabilization described by Joyce et al. are incorporated. Thus, DSCav1 is not the best in class immunogens for RSV, and this needs to be corrected and discussed.

We accept the view that other experimental vaccines have subsequently shown superior immunogenicity compared to DSCav1 in mouse immunogenicity studies. However, we have also directly compared, relative to the antigen contained within GSK’s recently licenced vaccine, Arexvy^TM^, which has demonstrated efficacy of 94% protection against severe disease during the first season and 67% throughout 3 seasons. As one of 2 licenced subunit vaccines for RSV and the only vaccine to date to demonstrate continued efficacy throughout 3 seasons, we consider this antigen to be the most relevant comparator for inclusion in our studies.

Similarly for hMPV, the comparator used is significantly less immunogenic than the triple disulfide versions, which were described in Ou et al. 2023 Plos Pathogens. Strangely, one of the most triple-disulfide comparators is shown in Table 1 (V3B-A12_D454C-V458C) but is this material is not compared immunogenically.

The results presented in Ou et al. 2023 did not directly compare V3B-A12_D454C-V458C to DS-CavEs2 and we are unaware of data indicating DS-CavEs2 to be less immunogenic as indicated by this reviewer. In fact, the results within Ou et al. 2023 show that V3B-A12_D454C-V458C elicits a comparable neutralising response to post-fusion hMPV-F, whereas the results of Hseih et al. (2022) show that DS-CavEs2 elicits a superior neutralising immune response over to post-fusion hMPV-F. We therefore believe that we have included a best-in-class comparator as indicated in the manuscript.

Bottom line: the author misrepresent their developed immunogens as being better than best-in-class comparators. The comparators chosen are not best-in-class. It would be optimal for the authors to carry out immunogenicity with best-in-class comparators.

Reviewer #3: The novelty of the manuscript is the use of a novel type of trimerization domain. This novel domain is likely another type of six-helix bundle of a non-human pathogen, but it is not described in the manuscript nor is there a reference to another paper. Therefore, this domain and the selection of the particular domain should be described in the paper. The design and the addition of glycans cannot be reviewed since there are no details.

We have now included a reference to the patent which includes these details.

The reduction of immune response against the silenced trimerization domain was two-fold. Although the response is lower compared with the non-silenced version, immune responses will increase after repeated vaccinations and the anti-MC2S will likely become more dominant after repeated vaccinations. Understandably, the authors want to highlight the novel trimerization domain, but they should not neglect earlier studies of pneumovirus vaccines without trimerization domains and include this in the discussion.

We have now included a sentence within the discussion to highlight this alternate approach (line 562-568); *‘It was recently demonstrated that further stabilisation of the RSV preF ectodomain can facilitate the omission of a trimerization domain thereby eliminating any off-target immune response from this domain ^50^. We also recognized the importance of reducing the off-target immune response, and achieved this through the incorporation of multiple N-linked glycosylation sites into MC2S. This strategy provides the benefit of easy purification process without drawback of off target response.’*

The authors write that comparable antigen homogeneity and oligomeric characteristics were conferred by MC and MC2 in Supplementary figure 2. However, in all cases except for influenza HA, the retention time is significantly shorter. How can this difference be explained? Has the Mw been analysed using SEC-MALS?

SEC results are overlayed from analysis conducted at different times and therefore we expect that these differences are due to run to run variation and differences in column compaction. We have now included the following sentence with the legend of Figure S2; ‘*Note. Slight differences in elution volumes are likely due to inter-run variation and column compaction.’*

Why does the large SARS2 spike protein have a longer retention time compared with Nipah, RSV and influenza proteins?

A different column (superose) was used for the SARS-CoV-2 due to the larger molecular weight. This detail is present in the figure and legend.

Why is MC2 only superior to MC for the influenza HA?
We do not have an explanation for this result. While this may form part of future studies within our platform, we have not repeated this result here as influenza is not our primary target.

The authors write that VXB-213 was engineered to include the optimal disulfide bridge linkages and furin cleavage site deletion identified by Joyce et al. Since this design is an optimized version of DS-Cav1, the authors should include text and reference to DS-Cav1 design (McLellan, 2013). VXB-213 also includes L373R. Could the author explain the origin and contribution of this mutation?

We have now included this reference within the results section (line 209/210); ‘VXB-213 was engineered to include the optimal disulfide bridge linkages and furin cleavage site deletion identified by McLellan *et al.,* ^33^ and Joyce *et al.,* ^34^,’

L373R is present in SC9-10DSCavAY.

Line 208: VXB-221 does not include an (unmodified) furin cleavage site. It contains a TMPRSS2 site which is not cleaved in the expression system used.

We have made this correction.

The description of DsCavEs2 in Table 1 is incomplete. DsCavEs2 also includes disulfides 110-322, 140-147, and it contain mutation E453Q.
Could the authors explain this omission? Does it mean the description is wrong? or is the naming wrong? In the last case, the authors have not compared immunogenicity of VXB-221 with a published variant, but with a protein of unknown characteristics and stability. The description of v3B_delta12 is correct and would have been a better comparator in the immunogenicity study based on the stability and other characteristics.

As also noted in the response to reviewer 1, we have gone back to our primary sequence data and confirmed that the correct sequence for DSCavEs2 was used. The mistake was introduced during drafting of table 1 for the document. This error has now been fixed in the resubmission.


Table 1 describes that hMPV Fsol is a Postfusion control. However, there is no evidence that the soluble F is transformed to postfusion. Please include evidence or change description. The publication of Battles et. al., (2017) shows how a postF protein can be generated.

The reviewer is correct, to ensure both RSV Fsol and hMPV Fsol were fully in the post-fusion state prior to immunisation they were heated to 70C for 2 hrs. We have now included this detail within results (line 289-290); *‘To ensure RSV and hMPV Fsol antigens were fully in the post-fusion state they were first heated to 70°C fo 2 hrs.’*

No superiority conclusions can be drawn from Ds-Cav1 and VXB-213 antigens, nor the DsCavEs2 vs VXB-221 antigens based on the ELISA or the mouse immunogenicity studies. Especially because the purification strategy does not allow such conclusions. The relatively unstable DsCav1 is purified using immunoaffinity strategies for which relatively harsh elution conditions are needed. Moreover, elution from 101F is not similar as elution from AVI8740. Apart from the sensitivity of the prefusion stability, based on supplementary Figure 6 (move this figure to main figures), the authors have not used the optimal purification method for DsCav1 and DsCavEs2 based on impurities shown in the gel, especially for DsCav1. Animal experiments can only be compared if purity is similar. Proteins should have been purified with additional SEC purification.

We have now included an acknowledgement that differences in the methods used to produce comparator antigens is a potential limitation within both the results and discussion sections; (line242-244), *‘This observation differs from the results reported by Hsieh et al., ^23^, where hMPV DSCavEs2 was expressed in Freestyle 293-F cells, in the presence of co-transfected trypsin and an additional SEC purification step.’* (line 527-529), *‘… however it should be noted that differences in purification methodologies used here and those used by Hseih et al., ^23^ may have contributed to this difference. ‘*

Please note that low pH elution from 101F was found to be detrimental to antigen conformation and so we used high pH for elution, which did not affect binding of pre-fusion specific antibodies. Also please note that comparison to Arexy is also included, which completely avoids such issues.

Moreover, if authors want to compare the vaccines, a dose titration study should be done instead of just one concentration.

Two dose levels were included in the comparison with Arexvy.

It is the stabilizing mutations that determine the stability of the prefusion conformation which is important for vaccine efficacy. Since for RSV and HMPV preF, (Ds-Cav1 vs VXB-213 and DsCavEs2 vs VXB-221) the stabilization strategy and the purity is different, it is not possible to draw conclusions on trimerization domain.
Since the novelty of the manuscript is the novel trimerization domain, not only the purity of the vaccines should be the same, but also the stabilizing substitutions should be identical between the foldon-fused and the MC2S-fused immunogens.

We accept the point that stabilization in the prefusion conformation is substantially mediated by the point mutations and the independent contribution of MC2S to such stabilisation has not been demonstrated. We have modified the text to focus on the lead antigens identified and removed claims that imply stabilisation is due to molecular clamp. Specifically, we have removed the use of the phrase ‘MC2S stabilised…’ in the following places; abstract line 49, results line 200, 238, discussion line 490, 581. In light of this change we do not believe it is necessary to directly compare the same antigen with MC2S and foldon.

Line 277: why was hMPV Fsol not used for depletion studies? And how is it possible that one animal immunized with Fsol has the highest PRNT50 titer of all animals?

This is an unexpected result, however it is not uncommon to have outliers in biological experiments. We do not have a specific explanation, but all results have been validated through an internal quality system and the neutralisation titre in this animal is real.

Line 351: DS7 is a bad choice if the authors want to catch the native prefusion conformation since DS7 recognizes a postF epitope.

We accept this view, however in our analysis and those in Williams et al., (2007), it appears that DS7 has affinity for both the pre and post-fusion conformations (although stronger for post-fusion) and is able to neutralise HMPV. We believe that the ability of DS7 to recognise preF is likely due to inherent flexibility in aa431-451, however this is out of scope for this publication and has not been discussed.

For the comparison of VXB-213 with Arexvy, the lyophilized proteins was reconstituted with PBS containing 0.05% PS80. For Arexvy, the adjuvant is part of the diluent, and from the description of Abrysvo it appears to be water. So both vaccines are not dissolved according to the instructions for an optimal formulation.
Were the immunisations with Arexvy performed with correctly reconstituted vaccine including the ASO1B adjuvant? Or was it PBS/tween reconstituted and mixed with Addavax?

To minimise the possibility of a negative effect on antigen conformation Arexvy was reconstituted in PBS+0.05% PS80 immediately prior to mouse vaccinations. This buffer was selected based on information included within the package insert. A further sentence has been added to the methods (line 672-674); ‘*Immediately prior to mouse vaccination, the Arexvy^TM^ antigen vial was resuspended in PBS containing 0.05% PS80 and then mixed with AddaVax (InvivoGen).’*

Line 441: Furthermore, the second generation MC2S also enhances a variety of antigen characteristics, such as conformational stability and yield relative to the first-generation MC (data not shown).
If not shown and no reference, this line should be deleted.

Deleted.

The comment in line 481 that VXB-213 could elicit higher neutralization at lower dosage than a DsCav1-based vaccine is speculation. Pfizer's Pref 847 also showed a much stronger induction of VNT compared with DS-Cav1. However, human trials showed same level of protection.
Yes, this comment is clearly speculative. The clinical trial underway (ClinicalTrials.gov ID NCT06556147) will directly compare Arexvy with lower dosages of VXB-241. We eagerly await the findings which will be reported in Q2 2025.

Combination of vaccines will indeed be an important next step in respiratory vaccine development. It is not clear why the stable components of Abrysvo would not be suited for such combinations.

Within its current formulation Abrysvo is lyophilised it may be possible that these antigens can be combined with preF antigens from hMPV, or other viruses either lyophilised or in liquid formulation however such results have not been completed. Furthermore, the present technological approach is achieving combination of these antigen in fully liquid formulation which is certainly preferable for convenience of administration.

Since vaccines will be used in pre-immune population, a comparison in a pre-immune model would be interesting.

We agree that such results would be interesting but with the findings from the Phase 1 clinical trial due to be reported in Q2 2025, there is not time to conduct additional studies in animals prior to the much more relevant findings that will come from the human population.

The authors claim that the neutralizing antibodies against the internal trimer interface are potent. However, after the publication of Rapazzo et. al. (Immunity 2022), such antibodies can no longer be labeled as potent. Site 0 antibodies like ADI-61026 are now the benchmark of potent neutralizing antibodies.

We agree and have deleted the word potent from line 138.

References to the Icosavax press release don’t work. I am also not sure whether references to press releases should be used for scientific manuscripts.

We have deleted the reference to Icosavax press release. However, we believe this is worth mentioning as this is the only completed clinical trial for RSV/hMPV bivalent vaccine that we are aware of although unfortunately not yet within the peer reviewed literature.

For antigen – specific IgG responses, the antigens are coated directly on ELISA plates. This can cause denaturation and loss of epitopes.

We agree that this is a potential issue and have included additional wording in relation to this within the discussion (line 570-573); ‘… *by directly coating antigen to ELISA plates. It should however be noted that direct coating can cause antigen denaturation and so such results may underestimate antigen specific or domain specific IgG responses.’*

In line 510, a reference is made to a fusion inhibitor study of Battles et. al., (2016). This reference seems out of place. The Krarup (Nat Comm 2015) publication describes the dependency on full cleavage for RSV F trimerization.

Inclusion of the Battles et al (2016) reference was a mistake. This has now been corrected to Ou et al., (2023), which relates to the design of a closed prefusion antigen for hMPV.

Line 511 and beyond has too much speculation on the possible advantages of an open HMPV trimer vaccine and lacks discussion of recent insights.

While speculative, we believe this paragraph discusses a novel and relevant point of view and highlights the difference in the direction we have taken in design of hMPV preF antigen (open / flexible) vs that taken by others within the field (closed / highly stable).

**Part III – Minor Issues: Editorial and Data Presentation Modifications**
Please use this section for editorial suggestions as well as relatively minor modifications of existing data that would enhance clarity.

Reviewer #1: 1. Line 62: RSV was discovered in 1956, not 1957.

Corrected, thank you.

2. Supplementary Figure 2: The authors should provide an explanation for the shifted peaks observed between MC and MC2 antigens in panels A, B, and D, but not C. Clarify whether these shifts are expected or indicative of structural or conformational differences.

SEC results are overlayed from analysis conducted at different times and therefore we expect that these differences are due to run to run variation and differences in column compaction. In addition MC2 contains an additional n-linked glycan site relative to MC. We have now included the following sentence with the legend of Figure S2; ‘*Note: slight differences in elution volumes are likely due inter-run variation, the effect of column compaction, as well as MC2 containing an additional N-linked glycan site relative to MC.’*

3. Supplementary figure 3: Specify which dose(s) was/were used for animal immunizations. Currently, the description in line 626 ("Mice were immunized with 0.5 µg, 2 µg, or 3 µg per recombinant protein antigen stated") is unclear and makes interpretation of results difficult.

Additional information has been added into the figure legend; *‘Two intramuscular doses were administered to Balb/C mice three weeks apart with each 50µl dose containing either 5µg (SARS-CoV-2 S, Nipah F, Influenza HA), or 1µg (RSV F) of purified protein in PBS and 25µl of AddaVax (Invivogen). Three weeks following second dose blood serum was collected for analysis of virus neutralisation.’*

4. Lines 219-220: the sentence “RSV preF antigens, 94% aggregate, 0% trimer; hMPV preF antigen, 25% aggregate, 19% trimer, 56% monomer” is very vague and confusing. Please clearly specify which antigens are being discussed within the paragraph.

This sentence has now been corrected for clarity; *‘Both the RSV and hMPV antigen panels showed similar trends for their oligomerisation profiles, with the non-stabilised Fsol proteins producing the greatest proportion of aggregate (RSV Fsol, 94% aggregate, 0% trimer; hMPV Fsol, 25% aggregate, 19% trimer, 56% monomer), the MC2S-incorporating antigens showing high degrees of trimer homogeneity (>89% trimer for both VXB-213 and VXB-221) with the comparator antigens being intermediate to these two (RSV DSCav1, 69% trimer; hMPV DSCavEs2, 54% trimer).’*

5. The term "dissociation constant" is incorrectly typed as “kD” throughout the manuscript. Please replace with “KD”.
Corrected.

6. Line 285: the authors should clarify that “VXB-241” is a combination of VXB-213 and VXB-221 antigens.

Corrected.

7. Line 377: Based on Figure 3B, value “15.5%” seems to be a typo, as the values for Arexvy antigens in the plot are 19.1% and 19.2%. The authors should check for data accuracy.

Corrected.

8. Line 398–399: The statement “…potentially indicating some interference due to immunodominance of the RSV preF antigen” requires further clarification. Either provide supporting data or cite relevant literature, as there is currently no evidence included in the figures to substantiate this claim.

This sentence refers to the data presented in figure 3c and 3d. Here we see that for the bivalent formulation there is no change in the neutralisation level against RSV monovalent formulation whereas there is a drop in the neutralisation level against hMPV

Reviewer #2: (No Response)

Reviewer #3: (No Response)

PLOS authors have the option to publish the peer review history of their article ([what does this mean?](https://track.editorialmanager.com/CL0/https:%2F%2Fjournals.plos.org%2Fplospathogens%2Fs%2Feditorial-and-peer-review-process%23loc-peer-review-history/1/010f0194ac392116-151b8bea-74e0-4950-b27a-42d0ac360ccc-000000/RUnpRnlO9jil1oUbiLH1vT5L1ZCISzexmVUTN8_WX_k=195)). If published, this will include your full peer review and any attached files.


If you choose “no”, your identity will remain anonymous but your review may still be made public.

**Do you want your identity to be public for this peer review?** For information about this choice, including consent withdrawal, please see our [Privacy Policy](https://track.editorialmanager.com/CL0/https:%2F%2Fwww.plos.org%2Fprivacy-policy/1/010f0194ac392116-151b8bea-74e0-4950-b27a-42d0ac360ccc-000000/YBwHtErrvr7lOYJhr50CVddIau7T_8k6tL1PDe6MGvM=195).

Reviewer #1: No

Reviewer #2: No

Reviewer #3: No

[NOTE: If reviewer comments were submitted as an attachment file, they will be attached to this email and accessible via the submission site. Please log into your account, locate the manuscript record, and check for the action link "View Attachments". If this link does not appear, there are no attachment files.]

**Figure resubmission:**

While revising your submission, please upload your figure files to the Preflight Analysis and Conversion Engine (PACE) digital diagnostic tool, https://pacev2.apexcovantage.com/. PACE helps ensure that figures meet PLOS requirements. To use PACE, you must first register as a user. Registration is free. Then, login and navigate to the UPLOAD tab, where you will find detailed instructions on how to use the tool. If you encounter any issues or have any questions when using PACE, please email PLOS at figures@plos.org. Please note that Supporting Information files do not need this step. If there are other versions of figure files still present in your submission file inventory at resubmission, please replace them with the PACE-processed versions.

**Reproducibility:**

To enhance the reproducibility of your results, we recommend that authors of applicable studies deposit laboratory protocols in protocols.io, where a protocol can be assigned its own identifier (DOI) such that it can be cited independently in the future. Additionally, PLOS ONE offers an option to publish peer-reviewed clinical study protocols. Read more information on sharing protocols at https://plos.org/protocols?utm_medium=editorial-email&utm_source=authorletters&utm_campaign=protocols
