# Supplementary material for: A second-generation molecular clamp stabilised bivalent candidate vaccine for protection against diseases caused by respiratory syncytial virus and human metapneumovirus
Source: PLoS Pathog. 2025 Jul 17;21(7):e1013312. doi: 10.1371/journal.ppat.1013312 (PMC12270161; doi:10.1371/journal.ppat.1013312)
Supplement: Attachment — Submitted filename: Response to second review.docx [file ppat.1013312.s012.docx]

**Reviewers' Comments:**

Reviewer's Responses to Questions

**Part I - Summary**
Please use this section to discuss strengths/weaknesses of study, novelty/significance, general execution and scholarship.

Reviewer #1: The authors have generally addressed my prior comments, and changes to the resulting text have improved the accuracy of those statements. It is a pity though that comparisons to previously reported antigens, since as Ds-CavEs2, are still difficult to interpret given that the authors did not express and purify the antigens according the published procedures. There is presumably a reason why co-transfection with a plasmid containing furin and SEC purification was performed in Hsieh et al. At least now the authors of this manuscript acknowledge that some of the differences observed in their studies could be due to different expression and purification strategies.

Reviewer #2: Chappell and colleague describe an interesting trimeric molecular clamp, derived the post-fusion HIV-1 gp41 six-helix bundle, which they utilize in vaccines against respiratory syncytial virus and human metapneumovirus. The manuscript has a lot of nice data, but has suffered from “overclaiming”, as indicated by comments from the reviewers. These substantially detracted from an otherwise nice paper.

As outlined below we have now removed further instances of overclaiming and further included

Reviewer #3: -

**Part II – Major Issues: Key Experiments Required for Acceptance**
Please use this section to detail the key new experiments or modifications of existing experiments that should be absolutely required to validate study conclusions.

Generally, there should be no more than 3 such required experiments or major modifications for a "Major Revision" recommendation. If more than 3 experiments are necessary to validate the study conclusions, then you are encouraged to recommend "Reject".

Reviewer #1: N/A

Reviewer #2: (No Response)

Reviewer #3: The authors have included acknowledgement that differences in the methods used to comparator antigens is a potential limitation, but they only describe this for the HMPV immunogen and not for the 101F based purification of RSV immunogens. The limitation of the purification of the latter should also be included irrespective of the fact that another experiment was done with the commercial vaccine as a comparator.

We have now included an additional sentence highlighting this limitation. Line 240-242 *‘Similarly, we cannot exclude the possibility that RSV DSCav1 produced here in expiCHO-S (Thermofisher), may be conformationally different to that produced by McLellan et al., in HEK293K cells ^31^.’*

The comment that VXB-213 could elicit higher neutralization at lower dosage than a DsCav1-based vaccine is speculation. The authors agree and mention that the clinical trial is underway but they did not delete the speculative sentence. This should be deleted and authors should wait for the trial data to make this claim.

We have now deleted these sentences (lines 497-500).

Reviewer mentioned that in line 511 and beyond (first version) there was too much speculation on the possible advantages of an open HMPV trimer vaccine and lacks discussion of recent insights.
Authors agree that it is speculative but decided not to change the text while this section is still too speculative and should be toned down. If the authors insist to discuss so much details on this subject and if they think it is relevant, they should mention that the anti-interface antibodies are far less potent compared with the site 0 antibody ADI-61026

We respectfully disagree with the reviewers statement that anti interface antibodies are ‘far less potent compared with the site 0 antibody ADI-61026’. Rappazzo et al., 2022 DOI: [10.1016/j.immuni.2022.07.003](https://doi.org/10.1016/j.immuni.2022.07.003) reports the neutralising titre (IC50) of ADI-61026 to be 12ng/ml against hMPV-A2 whereas Huan et al., 2020 DOI: [10.1371/journal.ppat.1008942](https://doi.org/10.1371/journal.ppat.1008942) reports the neutralizing titre of interface specific mAb MPV-458 to be 33ng/ml.

We do however accept that interface targeting mAbs have variable neutralising potency and so as way of compromise have included the following sentences (line 542-547) *‘However, we note that monoclonal antibodies targeting the internal interface of the hMPV F protein exhibit varying levels of neutralizing potency. While some, such as M8C10 and MPV465, show modest neutralizing activity, others like MPV-458 demonstrate both high neutralizing potency and cross-reactivity across hMPV strains, highlighting the potential importance of this conserved epitope for vaccine design ^28,50^.’*

**Part III – Minor Issues: Editorial and Data Presentation Modifications**
Please use this section for editorial suggestions as well as relatively minor modifications of existing data that would enhance clarity.

Reviewer #1: N/A

Reviewer #2: While the authors have done a decent job responding to reviewers, and the manuscript is improved, there are still overstatements that should be removed.

For example, in the abstract, the authors have not modified their best-in-class comparative claim that “Head-to-head evaluations in mouse immunogenicity studies showed that the VXB-241 candidate vaccine induced a neutralizing immune response that was superior or equivalent to the best-in-class comparator antigens…” As indicated in my prior review, it’s inappropriate to call the DS-Cav1 immunogen a “best-in-class comparator antigen” when discussing immunogenicity, as there are other described antigens with higher immunogenicity, including those from Joyce et al, with more than 3-fold increased immunogenicity in mice than DS-Cav1.

We have deleted ‘best-in-class’ and replaced with ‘prefusion stabilised’ (line 54).

The authors counter in their response that DS-Cav1 is the best-in-class, since the clinical trial results are so good. But what is being compared in this paper is mouse immunogenicity, not clinical trial results. Thus, it’s critical that the authors do not call DS-Cav1 ‘best-in-class’, when referring to immunogenicity data. In truth, there wouldn’t be much difference to the abstract to merely removed the “best-in-class” state, e.g., “Head-to-head evaluations in mouse immunogenicity studies showed that the VXB-241 candidate vaccine induced a neutralizing immune response that was superior or equivalent to comparator antigens…” This accurately states the data but does not overclaim.
as above

Also, since the MC2S is the focus of this paper, it might be helpful to have a figure showing the explicit modifications that were introduced; such a figure could easily be added to the supplemental, enabling interested readers to immediately see this important part of the manuscript.
We believe this is clearly laid out in table 1 of the main document. We can include a figure for this in the supplementary but believe this unnecessary and adds little to the general reader.

Lastly, since MC2S is a trimerization “clamp” based on helical interactions, the authors should add a few sentences to the discussion comparing MSC2 to other trimeric stabilizations, such as the trimer-adapted version of GNC4, which as been used to stabilized other prefusion trimeric immunogens as well as the helical zipper derived from RSV itself (Stewart-Jones, et al., 2015 “A Cysteine Zipper Stabilizes a Pre-Fusion F Glycoprotein Vaccine for Respiratory Syncytial Virus”).

We have now included a sentence in the discussion that the MC platform is a trimerization domain similar to previously utilised alternatives GCN4 and Foldon. Line 454-455: *‘The MC platform is essentially a high stability trimerization domain, similar to previously utilised alternatives GCN4 and Foldon ^45,46^.’*

Reviewer #3: -

PLOS authors have the option to publish the peer review history of their article ([what does this mean?](https://track.editorialmanager.com/CL0/https:%2F%2Fjournals.plos.org%2Fplospathogens%2Fs%2Feditorial-and-peer-review-process%23loc-peer-review-history/1/010f0196ee44b211-2ab2322c-cf93-4104-890e-46b50953cca4-000000/WlOJosIgvTIR61o7Ac8aMaOHfAFbvyP-augB3YQuRJE=211)). If published, this will include your full peer review and any attached files.

If you choose “no”, your identity will remain anonymous but your review may still be made public.

**Do you want your identity to be public for this peer review?** For information about this choice, including consent withdrawal, please see our [Privacy Policy](https://track.editorialmanager.com/CL0/https:%2F%2Fwww.plos.org%2Fprivacy-policy/1/010f0196ee44b211-2ab2322c-cf93-4104-890e-46b50953cca4-000000/_gMxQtriGIj8NiSChEYO6Kre2jDfr1DrEADJMcNp_H8=211).

Reviewer #1: No

Reviewer #2: No

Reviewer #3: No

Additional change: As the Icosavax IVX-A12 clinical trial has now been published we included this reference and data. Line 129-131: *‘Icosavax, now part of AstraZeneca, is also developing a bivalent RSV-hMPV vaccine (IVX‑A12), based on virus-like particles (VLPs), which has been demonstrated to boost neutralsing antibody titres against both RSV and hMPV by 3-5 fold ^20^.*
